# Aural Foreign Bodies: Descriptive Study of 224 Patients in Al-Fallujah General Hospital, Iraq

**DOI:** 10.1155/2013/401289

**Published:** 2013-12-03

**Authors:** Ahmad Nasrat Al-juboori

**Affiliations:** Ibn Sina College of Medicine, Al-Iraqia University, Baghdad, Iraq

## Abstract

Foreign bodies (FB) in the external auditory canal are relative medical emergency. The objective of this study was to describe the types of FB and their complications and to highlight on new FB not seen before which was the bluetooth devices that were used for cheating during high school examination in Al-Fallujah city. This was a two-year hospital-based descriptive study performed in the Department of Ear, Nose and Throat (ENT), Al-Fallujah General Hospital, from June 2011 to May 2013; during this period, 224 FB had been extracted from 224 patients. Beads were extracted from 68 patients (30.4%), cotton tips were extracted from 50 patients (22.3%), seeds and garlic were extracted from 31 patients (13.8%), papers were extracted from 27 patients (12.1%), insects were extracted from 24 patients (10.7%), button batteries were extracted from 13 patients (5.8%), and bluetooth devices were extracted from 7 patients (3.1%). Most of the cases did not develop complications (87.5%) during extraction. The main complications were canal abrasion (4.5%). Proper instrumentation allows the uncomplicated removal of many FB. The use of general anesthesia is preferred in very young children. Bluetooth device objects should be considered as new aural FB, especially in our territory.

## 1. Introduction

Foreign bodies (FB) in the external auditory meatus are most commonly seen in children who have inserted them into their own ears. Children may present asymptomatically, or with pain or a discharge caused by otitis externa. Adults are often seen with cotton wool or broken matchsticks which have been used to clean or scratch the ear canal [1]. Live insects in the ear, commonly small cockroaches [2], are annoying due to discomfort created by loud noise and movement. FB in the ear is relatively common in emergency medicine. However, attempts of removal made outside the healthcare setting by untrained persons can result in complications of varying degrees [3]. An aural FB can involve damage to tympanic membrane or middle ear by itself or by improper management during removal. The etiology of FB in the ear has been ascribed to general curiosity and a whim to explore orifices in children, playful insertion of FB into others' body parts, accidental entry of foreign body, preexisting disease in ear causing irritation, and habitual cleaning of ear and nose with objects like ear buds [4, 5]. FB in ear can be classified in many ways like organic-inorganic, animate-inanimate, metallic-nonmetallic, hygroscopic-nonhygroscopic, regular or irregular, soft or hard, and so forth, according to their nature [6]. The method of removal usually depends on the type of FB, its position, and cooperation of the patient [7, 8]. Based on criteria used by American Family Physician (with Strength of Recommendation Taxonomy (SORT) grade C), all ear FB cases should be referred to ENT specialty for removal except for only those which are directly visible and “graspable” [9].

The objective of this study was to describe the types of FB and their complications and to highlight on new FB not seen before which was the bluetooth devices that were used for cheating during high school examination in Al-Fallujah city.

## 2. Materials and Methods

This was a two-year hospital-based prospective descriptive study performed in the Department of Ear, Nose and Throat (ENT), Al-Fallujah General Hospital, from June 2011 to May 2013; during this period, 224 FB had been extracted from 224 patients. History and patients data included age, sex and presenting symptoms had been taken as well as ear, nose and throat examination was performed. All patients with suggestive history of FB entry into ear were included. Those patients with no suggestive history but were found to have the FB are also included in the study. Patients with complications arising out of FB, whose extraction was done at a different centre, are excluded. The use of aural syringing, vacuum suction, and manual instrumentation by the use of Jobson Horne's probe or hook and forceps may be indicated. In a very limited number of patients, especially in children, general anesthesia was used because of poor cooperation. After extraction of FB, reexamination of the affected ear was performed immediately and after three days to exclude the possible complications.

## 3. Results 

The total number of patients with FB was 224 patients; they ranged from below one year to above 60 years old, and the mean age with standard deviation was 19 years ±2.1 years. They were 139 male patients and 85 patients were females, with male to female ratio of 1.6 : 1 as shown in Table 1. The onset of presentation was noticed mainly in the first 24 hours of the injury; 180 of such patients presented in the first 24 hours, 25 patients presented in the second 24 hours, and 13 patients presented between 48 and 72 hours of the onset, while the remaining six patients presented after 72 hours.

The types of 224 aural FB extracted from the patients are shown in Table 2 in order of frequency. Beads extracted from 68 patients (30.4%), cotton tips extracted from 50 patients (22.3%), seeds in different types and garlic extracted from 31 patients (13.8%), papers extracted from 27 patients (12.1%), insects extracted from 24 patients (10.7%), button batteries extracted from 13 patients (5.8%), bluetooth devices extracted from 7 patients (3.1%), and miscellaneous types of FB including matchstick, eraser, and stone are shown in Table 2.

Bluetooth device objects were used in cheating during students' examinations, especially college and secondary school students, where the concealed mobile device was used and bluetooth metallic pieces were applied in contact with tympanic membrane, with the help of another person present outside the examination hall test for the purpose of the solving questions. Those aural foreign bodies were not seen before.

Here, the insertion of these magnetic bluetooth device objects done by someone else, who inserted them inside the ear canal (Figure 2), and after the end of the examination, they could not get rid of them; that is, they could not extract these objects from the ear canal, so that they consulted ENT clinic for extraction.

The complications which happened were observed due to presence of FB and/or during and after the extraction shown in Table 3. Most of the cases did not develop complications (87.5%). The main complications were canal abrasion (4.5%), canal laceration and/or bleeding (3.6%), otitis externa (3.1%), tympanic membrane perforation (0.9%), and otitis media (0.4%).

## 4. Discussion 

**Figure 1:**
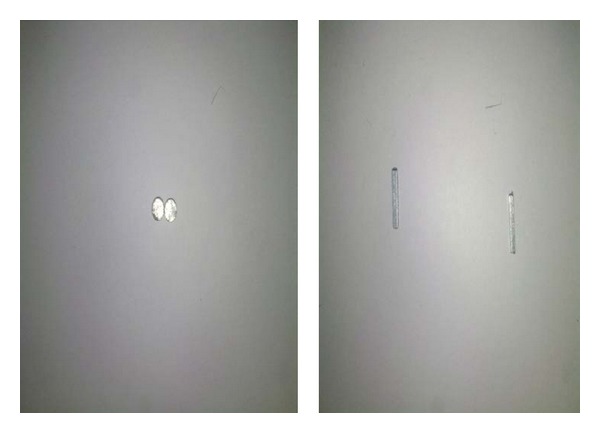
Different types of bluetooth devices extracted from the external auditory canal.

**Figure 2:**
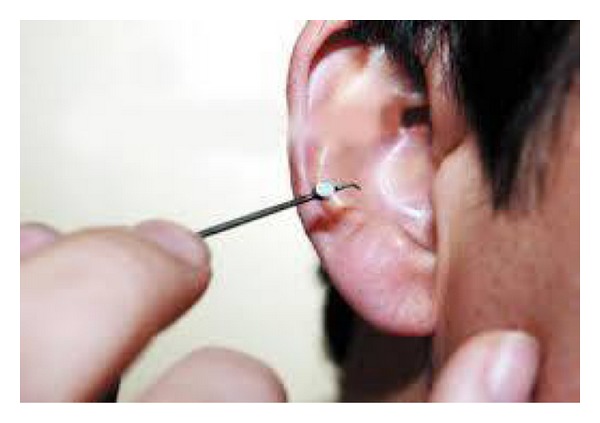
The method of inserting the bluetooth device in the external auditory canal.

Foreign body insertion into the ear in children is becoming increasingly common in developing countries. Children tend to be curious and exploratory; hence, the easily accessible orifices tend to be at risk of this form of injury [10]. In our study, the main age group below 10 years of age, representing 25.5%, was mostly affected; this was consistent with other studies. [3, 11]. A total of 480 cases were presented with ear FB during the study of Chai et al. The highest incidence of ear FB occurred in 0–5 years of age which consisted of 232 (48.3%) cases. This was followed by children between 6 and 10 years [12]. Most of the cases presented in the first 24 hours of the FB insertion, as in our study; this was also observed in other studies [3, 13]. There were wide variations regarding the type of the aural FB; in Chai et al. study, seeds or nuts were the commonest ear FB encountered which consisted of 226 (47.1%) cases; this was followed by plastic toys or beads [12]. In Ologe et al. study grains and seeds (27.9%), beads (19.7%), cotton wool (13.6%), paper (8.8%), and eraser (8.2%) formed the bulk of the aural FB [14], but this differed from our results in which beads and cotton tips were common as compared to seeds; this was consistent with other studies [15]. In our study, garlic was encountered as an animate FB because it was used traditionally for the relief of earache. Bluetooth device objects were small pieces of magnetic property (Figure 1) used with the aid of mobile for cheating during final examinations in high school; this was one of the figures of corruption; this metallic piece was introduced through the ear canal and applied in contact with the tympanic membrane (Figure 2). Here, there was a person outside the examination hall answering the key questions and sending the solution to the examiner; this type of FB was not recorded or mentioned before, but we recorded seven cases after the insertion of those small objects in the external auditory canal (EAC).

Complication due to presence of FB or the extraction was uncommon; no complications were recorded in 87.5% of the cases in contrast to Singh et al. study which recorded 77% complication rate [16]. Adequate immobilization and proper instrumentation allow the uncomplicated removal of many EAC foreign bodies in the pediatric population. The use of general anesthesia is preferred in very young children and in children of any age with aural FB whose contour, composition, or location predispose to traumatic removal in the ambulatory setting [17].

## 5. Conclusion 

Proper instrumentation allows the uncomplicated removal of many EAC foreign bodies. The use of general anesthesia is preferred in very young children and the uncooperative. Bluetooth device objects should be considered as new aural FB, especially in our territory.

## Figures and Tables

**Table 1:** Age and sex distribution.

Age (years)	Male	Female	Total (%)
<1–10	37	20	57 (25.5)
11–20	31	13	44 (19.6)
21–30	19	18	37 (16.5)
31–40	20	16	36 (16.1)
41–50	21	10	31 (13.8)
51–>60	11	8	19 (8.5)

Total	139	85	224 (100)

**Table 2:** Types of aural foreign bodies extracted from 224 patients.

Types of foreign body	Number	Percentage
Beads	68	30.4
Cotton tips	50	22.3
Seeds and garlic	31	13.8
Paper	27	12.1
Insects	24	10.7
Button batteries	13	5.8
Bluetooth device	7	3.1
Miscellaneous*	4	1.8

Total	224	100

*Matchstick, eraser, and stone.

**Table 3:** Complications of aural foreign body extraction from 224 patients.

Complications	Number	Percentage
No complications	196	87.5
Canal abrasion	10	4.5
Canal laceration and/or bleeding	8	3.6
Otitis externa	7	3.1
Tympanic membrane perforation	2	0.9
Otitis media	1	0.4

Total	224	100

## References

[1] Kroukamp GR, Loock JW, Gleeson M, Browning GG, Burton MJ (2008). Foreign bodies in the ear. *Scott-Brown's Otorhinolaryngology, Head and Neck Surgery*.

[2] Kroukamp G, Londt JGH (2006). Ear-invading arthropods: a South African survey. *South African Medical Journal*.

[3] Olajide TG, Ologe FE, Arigbede OO (2011). Management of foreign bodies in the ear: a retrospective review of 123 cases in Nigeria. *Ear, Nose and Throat Journal*.

[4] Das SK (1984). Aetiological evaluation of foreign bodies in the ear and nose (a clinical study). *Journal of Laryngology and Otology*.

[5] Kalan A, Tariq M (2000). Foreign bodies in the nasal cavities: a comprehensive review of the aetiology, diagnostic pointers, and therapeutic measures. *Postgraduate Medical Journal*.

[6] Schulze SL, Kerschner J, Beste D (2002). Pediatric external auditory canal foreign bodies: a review of 698 cases. *Otolaryngology—Head and Neck Surgery*.

[7] Balbani APS, Sanchez TG, Butugan O (1998). Ear and nose foreign body removal in children. *International Journal of Pediatric Otorhinolaryngology*.

[8] Thompson SK, Wein RO, Dutcher PO (2003). External auditory canal foreign body removal: management practices and outcomes. *Laryngoscope*.

[9] Mohamad I (2012). Ear foreign body: tackling the uncommons. *Medical Journal of Malaysia*.

[10] Ibekwe M, Onotai L, Otaigbe B (2012). Foreign body in the ear, nose and throat in children: a five year review in Niger delta. *African Journal of Paediatric Surgery*.

[11] Wada I, Kase Y, Iinuma T (2003). Statistical study on the case of aural foreign bodies. *Journal of Otolaryngology of Japan*.

[12] Chai CK, Tang IP, Tan TY, Jong DEYH (2012). A review of ear, nose and throat foreign bodies in Sarawak General Hospital. A five year experience. *Medical Journal of Malaysia*.

[13] Hon SK, Izam TM, Koay CB, Razi A (2001). A prospective evaluation of foreign bodies presenting to the Ear, Nose and Throat Clinic, Hospital Kuala Lumpur. *Medical Journal of Malaysia*.

[14] Ologe FE, Dunmade AD, Afolabi OA (2007). Aural foreign bodies in children. *Indian Journal of Pediatrics*.

[15] Ryan C, Ghosh A, Wilson-Boyd B, Smit D, O’Leary S (2006). Presentation and management of aural foreign bodies in two Australian emergency departments. *Emergency Medicine Australasia*.

[16] Singh GB, Sidhu TS, Sharma A, Dhawan R, Jha SK, Singh N (2007). Management of aural foreign body: an evaluative study in 738 consecutive cases. *American Journal of Otolaryngology*.

[17] Ansley JF, Cunningham MJ (1998). Treatment of aural foreign bodies in children. *Pediatrics*.

